# Can you avoid admitting patients with skin infections?

**DOI:** 10.1093/jacamr/dlz014

**Published:** 2019-04-12

## Abstract

**Graphical Abstract:**
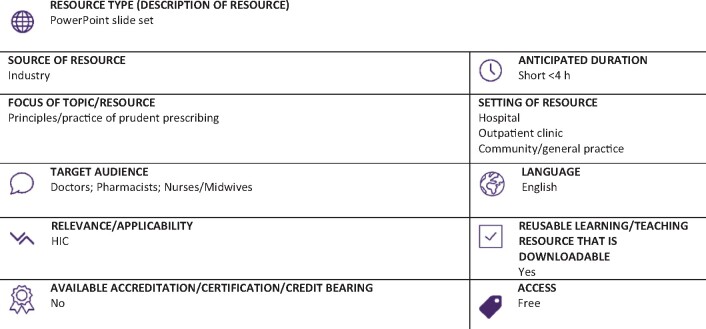

**Resource web link:** **http://bsac-jac-amr.com/wp-content/uploads/2019/04/XYD001_XYDALBA-Dalbavancin_SLIDES-FOR-Manchester-conference.pptx** (Full classification scheme available at: http://bsac.org.uk/wp-content/uploads/2019/03/Educational-resource-review-classification-scheme.pdf)

**WHO region and country (World Bank):** Europe, UK (HIC)

## Peer review commentary

This resource is an industry-prepared (Correvio) PowerPoint slide set about the use of Dalbavancin, a long-acting lipoglycopeptide, for the treatment of acute skin and soft tissue infections (aSSTI).

The 36 slides comprise an introduction to factors to consider when treating aSSTI infections and considerations for the use of Dalbavancin for outpatient parenteral antimicrobial therapy (OPAT).

The resource provides a comprehensive overview of the use of Dalbavancin alongside clinical trial data (two large, randomized trials designed to demonstrate non-inferior efficacy compared with vancomycin/linezolid). The slide set provides evidence that a one and two-dose regimen of Dalbavancin provides a full course of treatment, with no requirement to adjust the dose or increase monitoring.

This resource would be a useful introduction for those healthcare practitioners who are looking for alternative treatment options for aSSTI; however, as this resource has been prepared by Correvio, the practitioner would be recommended to review other independent sources of evidence.

